# Effects of Multicomponent Training Followed by a Detraining Period on Frailty Level and Functional Capacity of Older Adults with or at Risk of Frailty: Results of 10-Month Quasi-Experimental Study

**DOI:** 10.3390/ijerph191912417

**Published:** 2022-09-29

**Authors:** Ángel Iván Fernández-García, Ana Moradell, David Navarrete-Villanueva, Jorge Subías-Perié, Jorge Pérez-Gómez, Ignacio Ara, Marcela González-Gross, José Antonio Casajús, Germán Vicente-Rodríguez, Alba Gómez-Cabello

**Affiliations:** 1 GENUD (Growth, Exercise, Nutrition and Development) Research Group, University of Zaragoza, 50009 Zaragoza, Spain; 2 Faculty of Health and Sport Sciences (FCSD), Department of Physiatry and Nursing, University of Zaragoza, Ronda Misericordia, 5, 22001 Huesca, Spain; 3 Red Española de Investigación en Ejercicio Físico y Salud en Poblaciones Especiales (EXERNET); 4 Faculty of Health Sciences, Department of Physiatry and Nursing, University of Zaragoza, 50009 Zaragoza, Spain; 5 HEME (Health, Economy, Motricity and Education) Research Group, University of Extremadura, 10003 Cáceres, Spain; 6 GENUD Toledo Research Group, Universidad de Castilla-La Mancha, 45071 Toledo, Spain; 7 CIBER of Frailty and Healthy Aging (CIBERFES), Instituto de Salud Carlos III, 28029 Madrid, Spain; 8 ImFine Research Group, Facultad de Ciencias de la Actividad Física y del Deporte-INEF, Universidad Politécnica de Madrid, 28040 Madrid, Spain; 9 Centro de Investigación Biomédica en Red de Fisiopatología de la Obesidad y Nutrición (CIBERObn), Instituto de Salud Carlos III, 28029 Madrid, Spain; 10 Instituto Agroalimentario de Aragón—IA2—(CITA-Universidad de Zaragoza), 50009 Zaragoza, Spain; 11 Centro Universitario de la Defensa, 50090 Zaragoza, Spain

**Keywords:** aging, exercise, physical activity, physical function, physical performance

## Abstract

This study aimed: To analyze the effects of 6-month multicomponent training (MCT) and 4-month detraining on functional capacity and frailty among older adults with/at risk of frailty and to analyze the influence of frailty status on training and detraining adaptations. A total of 106 older adults (80.5 ± 6.0 years) were divided into a control (CON) or training group (TRAIN). The TRAIN performed a 6-month MCT (Eelder-fit), while CON continued their usual lifestyle. Functional capacity was assessed by the Short Physical Performance Battery (SPPB), while frailty was evaluated through Fried (FP) and the short version of the Frailty Trait Scale (FTS-5). Linear mixed models were performed to analyze group effects and to compare differences in changes within and between groups. TRAIN showed improvements in SPPB (3.2 ± 2.4), FP (−0.7 ± 1.3), and FTS-5 (−5.9 ± 5.8), whereas CON improved in SPPB (0.7 ± 2.9) and deteriorated in FTS-5 (2.8 ± 7.6) (all *p* < 0.05). Group effects favorable to TRAIN were found for all scales during this period (all *p* < 0.05). After detraining, TRAIN worsened in SPPB (−1.2 ± 2.7) and FTS-5 (4.1 ± 6.1) (both *p* < 0.05). No relevant differences were observed, accounting for frailty status between TRAIN subgroups. Eelder-fit improved the functional capacity and frailty of this population, whereas 4-months of detraining caused a drop of these variables except in FP.

## 1. Introduction

Living longer does not mean living better; in fact, the real challenge of today is healthy and sustainable aging. The aging of the global population is accompanied by a growing burden of health problems [1] among which is the decline of functional capacity. It can lead to frailty, which can be defined as a progressive age-related decline in physiological systems that results in decreased reserves of intrinsic capacity, which confers extreme vulnerability [2]. Frailty negatively affects quality of life [3], and increases the risk of suffer adverse events (i.e., falls, fractures, cognitive decline, disability, hospitalization or even death) [4].

To get a perspective of the socioeconomic consequences of the problem, the prevalence of frailty and prefrailty in the world population is about 12 and 47%, respectively [5]. In addition, the transition from robustness to frail may increase up to 101% the spending of care-related costs [6], representing an annual cost of €2476 per patient [7]. Thus, the identification of frailty in a feasible and accurate way has become a recurrent issue in this field [6]. In the past few years, several attempts have been made to improve classical instruments [8] as Fried phenotype (FP). García-García et al. recently developed the Frailty Trait Scale (FTS), an instrument that incorporates new relevant domains according to the most recent findings about the pathophysiology of the syndrome [9]. In another study, García-García et al. developed the FTS short form of 5 items (FTS-5) in an attempt to easily assess frailty [10]. Moreover, the Short Physical Performance Battery (SPPB) has demonstrated to be an efficient, effective and accurate way to measure functional capacity [11] which has also been extensively used for frailty screening and study [12,13].

For all of the above, frailty prevention and treatment has become a major public health challenge [14]. Experts in the field have proposed exercise as a potential way to prevent and treat frailty in community-dwelling older adults [15]. In particular, evidence points out that multicomponent training (MCT) programs are one of the most effective interventions [16]. MCT has been included in the recommendations of physical activity (PA) for older adults by the World Health Organization [17]. Although many studies have focused on the effects of MCT on physical fitness [18] and cognitive function [19], there is still some controversy about which MCT protocol is the best for improving or alleviating frailty in older adults [20]. Moreover, to the author’s knowledge, no study has evaluated the effects of exercise above frailty according to the frailty status of older adults.

In addition, regular exercise programs for older adults are usually temporally interrupted during holiday periods. It seems that 3-month detraining period is enough to cause a deterioration in the physical fitness of older adults [21,22]. Due to the physiological peculiarities and lifestyle associated with aging, it is very likely that the potential benefits achieved during training will be lost, even more rapidly, in older adults with or at risk of frailty. Nonetheless, to date, little is known about the effects of detraining on the functional capacity and frailty levels of this specific population [23,24].

Therefore, the main aims of the present study were: (1) to analyze the effects of a 6-month MCT program on frailty level and functional capacity of older adults with or at risk of frailty; (2) to examine the consequences of a 4-month detraining period on frailty level and functional capacity; and (3) to analyze the influence of frailty status (diagnosed by the Fried Phenotype: robust vs. prefrail-frail) on training and detraining adaptations in the functional capacity and frailty level.

## 2. Materials and Methods

### 2.1. Study Design and Participants

This non-randomized controlled trial was carried out between 2018 and 2020 within the framework of the EXERNET-Elder 3.0 project. The study was performed in accordance with the Helsinki Declaration of 1961 revised by Fortaleza (2013) [25] and the current legislation of human clinical research in Spain (Law 14/2007). The study protocol was approved by the ethics committee of the Hospital Fundación de Alcorcón (16/50). This study was registered in the electronic repository clinicaltrials.gov (reference number: NCT03831841). A detailed description of the methodology was previously published elsewhere [26].

In brief, participants were recruited from four health care centers and three nursing homes for non-dependent people from Zaragoza, Spain. People over 65 years of age screened as frail or pre-frail according to the SPPB thresholds [13,27], were included in the study (SPPB < 10 points). Detailed information about the performance of this battery is provided below. The exclusion criteria were cancer and/or dementia. Of the 110 older adults who met the inclusion criteria and agreed to participate in the study, only those who completed at least two evaluations were included in the sample (*n* = 106). A sample size calculation was carried out for a power of 80% and 5% alpha level and to reject the null hypothesis H0: μ1 = μ2. Assuming a medium–large effect size (f = 0.30) and a correlation among repeated measures of 0.5, a sample size of 68 (34 per group) would be needed. The sample was increased by 20% to consider possible losses during follow-up. Thus, the final sample was 86 (43 per group).

Participants were allocated by convenience into the control group (CON) or training group (TRAIN) to maximize training attendance according to participant’s preferences and availability. The TRAIN completed a supervised 6-month MCT followed by a 4-month detraining period in which they continued with their routine activities, whereas the CON followed their usual lifestyle during the whole course of the project but underwent identical testing to the TRAIN at baseline and follow-ups. Moreover, during the whole project, participants of both groups received three talks related to healthy habits in order to engage CON participants throughout the study, reducing the possible drop-off caused by multiple evaluation periods. The talks lasted 1-h and they were performed by a certified nurse, nutritionist and sport scientist. The topics were “functional capacity and frailty,” “nutritional recommendations for older adults” and “physical exercise recommendations for older adults.” All of them were delivered by a certified nurse, nutritionist, and sport scientist.

### 2.2. Evaluations

Both groups were evaluated at three different times. Baseline assessment was performed before the training period (M0). The second evaluation was carried out at the end of the 6-month exercise program (M6) to examine the effects of MCT, whereas the last assessment was done at 10 months from the beginning to determine the effects produced by the 4-month detraining period (M10).

Functional capacity: Functional capacity was evaluated using SPPB. This battery measures balance by means of the progressive Romberg test (ability to stand up for 10 s with feet positioned in three ways: with feet together (semi-tandem and tandem), gait speed (time to complete a 4 m walk at usual pace) and strength of lower limb (time to rise five times from a chair). Each test was scored from 0 to 4, with a total battery score of 12 points (pt) [27]. SPPB was also evaluated at the middle of the training program (3-months from baseline).

Frailty: Although all participants were screened at the beginning of the study as frail or prefrail with SPPB battery [13], frailty was also assessed through FP [28] and the FTS-5 [10].

FP criteria are based on five items: unintentional weight loss (more than 4.5 kg in the last year or ≥5% of body weight), self-reported exhaustion (felt especially tired during the last week), weakness (low grip strength (Jamar Preston, Jackson, MI, USA), slow usual gait speed (4.5 m) and low physical activity (less than 2 h walking per week for women and 2.5 h for men). When three or more of these items were met, the degree of frailty was reached, while only one or two items denoted pre-frailty [28]. FP criteria were also used in order to analyze the effect of frailty status on training and detraining adaptations of TRAIN subgroups. This classification divided participants into robust, frail or prefrail [28].

The FTS-5 was constructed with domains of the FTS with the best predictive ability [10]. Those five items were energetic balance or nutrition evaluated by body mass index (BMI), activity through the Physical Activity Scale for the Elderly questionnaire (PASE), nervous system with progressive Romberg test, strength measured by grip strength (Jamar Preston, Jackson, MI, USA) and gait speed assessed by usual pace in 4 m. Each item ranges from 0 to 10 according to the scoring criteria [10]. FTS-5 scores from 0 (totally robust) to 50 (totally frail). The range from 0 to 25 evaluates the path from robust to frailty and from 26 to 50, who are extremely frail.

Health-Related, Body Composition Measurements and Physical Activity Assessment: The complete set of studied variables during the project is available elsewhere [26]. Specifically, the batteries and questionnaires included in this report to describe the sample were as follows: Instrumental Activities of Daily Living Scale [29], Barthel Index [30], Mini Nutritional Assessment [31], and Mini Mental State [32].

Height was measured with a portable stadiometer with a 2.10 m maximum capacity and a 1 mm error margin (Seca, Hamburg, Germany). A bioelectrical impedance (TANITA BC-418MA, Tanita Corp., Tokyo, Japan) was performed to obtain the body weight (kg) and percentage of fat mass. BMI was calculated by dividing weight (kg) by squared height (m^2^).

PA was monitored at baseline with wrist-worn triaxial accelerometers (GENEActiv, Activinsights Ltd., Cambridge, UK) following the methodology used in previous studies of the same project [33]. Participants wore the device on the nondominant wrist for 7 consecutive days, including 2 weekend days. Only those with a minimum of 4 valid days including at least 480 min (8 h/day) of wearing time were included in the analysis. Non-wear time detection was evaluated in blocks of 30 consecutive min following the methods described by Van Hees et al. [34]

### 2.3. Multicomponent Training Program: Eelder-Fit

The technical content of the program is based on a specific literature review [15,35,36]. Details of the methodology of Eelder-fit MCT have already been published previously [26]. In brief, the training protocol consisted of a 6-month MCT of three supervised training sessions per week of 1 h duration each (10 min of warm-up, 35–40 min of main part exercises and 10–15 min of cool down). The first and third weekly sessions, called “Strength and Functional sessions,” were used to perform strength, power, static balance exercises and tasks that simulate daily living activities. The second weekly session, named “Endurance sessions,” was used to execute aerobic basic exercises such as walking, steps and stationary cycle in addition to agility, coordination and motor skill tasks. During the whole MCT, there was a progression of the training load to provide an adequate stimulus to induce adaptations. Moreover, in order to individualize exercises, each session was adjusted according to the participants’ characteristics and functional capacity at baseline, as recommended by previous studies [12]. Training periodization and methodology are shown in Table 1; Table 2, respectively, and are divided into different phases with specific objectives and a standardized framework. Trainers recorded the attendance of TRAIN participants. To increase participation, the three elders of each TRAIN group who achieved the greatest percentage of attendance received sports equipment as an award.

### 2.4. Statistical Analysis

Statistical analyses were completed using the Statistical Package for the Social Sciences v. 20.0 for Windows (SPSS, Inc., Chicago, IL, USA). Values of *p* < 0.05 were considered statistically significant for all tests.

Descriptive data are presented as mean and standard deviation (SD) or number of participants (*n*) and percentage (%), according to the nature of each variable. Student’s *t*-test and Chi-square test were used to analyze differences between CON and TRAIN at baseline for continuous and categorical data, respectively.

Three linear mixed models were performed to analyze the main effects of intervention in functional capacity and frailty level during training (M0–M6) and detraining periods (M6–M10) and also to evaluate the residual effects of training (M0–M10). The models combine withing-group and between-group comparisons at different time points. Changes in variables were obtained by subtracting the data from the last evaluation minus the value of the previous evaluation.

Finally, linear mixed models analyses were also used to compare the evolution in the studied variables of different TRAIN subgroups according to their frailty status [28] (frails and prefrails (FRA-PRE) vs. robust (ROB): frail and prefrail were pooled together given the small sample size of frails).

The models considered the maximum likelihood estimation and the best-fitting covariance structure. For comparisons, group (TRAIN vs. CON) or frailty status-condition (ROB vs. FRA-PRE), period and sex were included as fixed factors, participants as random factors and baseline values and age as covariates. The significance level for all the tests was set at *p* < 0.05. Since no differences were found in the baseline between the groups in age and sex, the analyses were conducted with men and women as a whole group.

## 3. Results

### 3.1. Descriptive Characteristics of the Sample

The baseline characteristics of the sample are shown in Table 3. The sample included those participants who had data for at least two evaluation periods. Except in heigh, in which TRAIN participants were taller (*p* < 0.05), there were no differences between groups in any of the variables included in the study. Regarding attendance, the average rate reached by TRAIN participants was 83.2 ± 10.6%.

### 3.2. Effects of Multicomponent Training Program and Detraining Period on Functional Capacity

**Figure 1:**
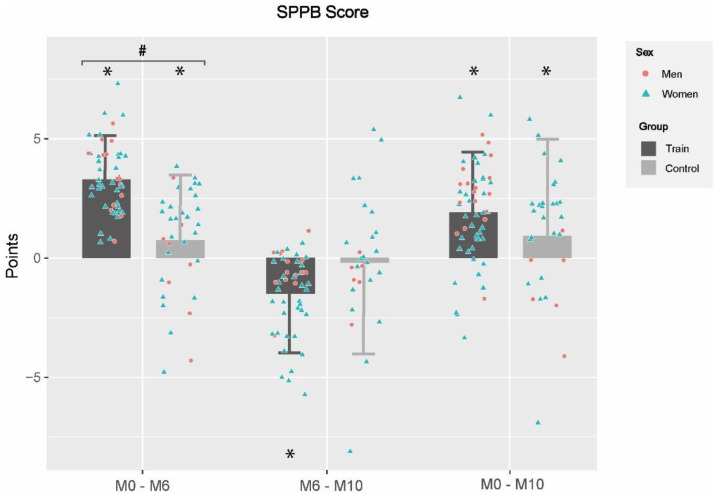
Changes in functional capacity between and within groups in different evaluation periods. M0–M6: changes between baseline and 6th month; M6–M10: changes between 6th and 10th month; M0–M10: changes between baseline and 10th month; CON: Control Group; TRAIN: Training Group; *: Statistical significance within-group changes; #: group effects. Differences were obtained by linear mixed models adjusted by baseline values, gender and age; statistical significance was set at *p* < 0.05.

Changes in functional capacity (SPPB score) are shown in Figure 1. When pre-training values were compared with post-training (M0–M6), both groups showed significant improvements in SPPB scores (TRAIN: 3.2 ± 2.4; CON: 0.7 ± 2.9). TRAIN improved significantly in every single test of the battery (Table 4), while CON also improved in the chair stand test and the 4m gait speed test, whereas they worsened in the Romberg test (all, *p* < 0.05). Group effects were found for SPPB and in all the test of the battery, being favorable to TRAIN (all, *p* < 0.001).

### 3.3. Effects of MCT Program and Detraining Period on Frailty Level

**Figure 2:**
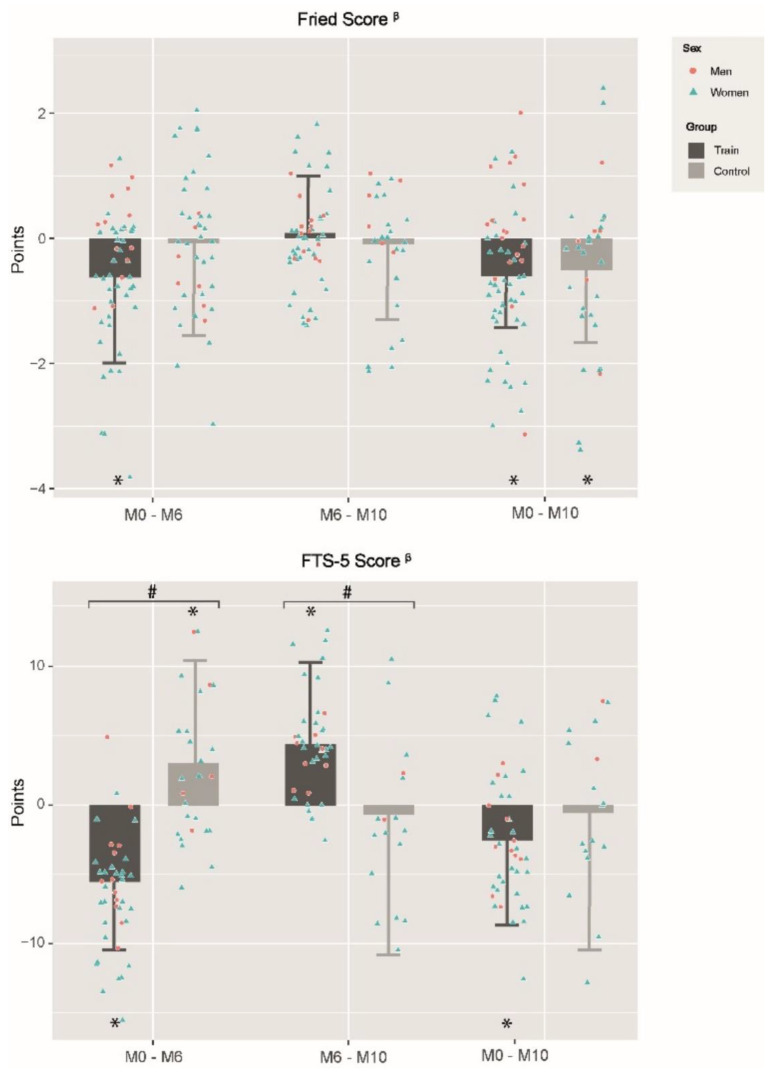
Changes in Fried’s Frailty Phenotype and FTS-5 between and within groups in different evaluation periods. M0–M6: changes between baseline and 6th month; M6–M10: changes between 6th and 10th month; M0–M10: changes between baseline and 10th month; CON: Control Group; TRAIN: Training Group; FTS-5: Frailty Trait Scale of 5-items; *: Statistical significance within group changes; #: group effects; ^β^: negative changes represent frailty diminution; Differences were obtained by linear mixed models adjusting by baseline values, gender and age; Statistical significance was set at *p* < 0.05.

Figure 2 shows the changes in frailty levels with FP and FTS-5, whereas the progression in the specific tests of FTS-5 is shown in Table 5. When pre-training values were compared with post-training (M0–M6), TRAIN significantly improved their frailty level above both scales, showing a lower score in FP (−0.7 ± 1.3) and FTS-5 (−5.9 ± 5.8) (*p* < 0.001). On the contrary, CON obtained a significant score increase in FTS-5 (2.8 ± 7.6) (*p* < 0.005). Furthermore, when different domains of FTS-5 were analyzed separately, CON showed worse values after post-training in the Romberg test and PASE. By contrast, TRAIN got better results not only in the Romberg test and PASE, but also in the 4m gait speed test and grip strength. Group effects were observed not only in FP, but also in FTS-5 and all its specific variables (*p* < 0.001), except in BMI and grip strength.

Regarding the changes observed when post-training results are compared with post-detraining (M6–M10), TRAIN worsened in FTS-5 (4.1 ± 6.1) and also in the Romberg test (−1.6 ± 4.8), PASE (−25.2 ± 41.8) and 4m gait speed test (0.7 ± 1.3) (all *p* < 0.05), while no significant changes were observed in CON. Group effects were found in the FTS-5 and 4 m gait speed (*p* < 0.001), which were both favorable to CON.

Finally, in the post-detraining evaluation, both groups improved in FP score and grip strength with respect to pre-training, and TRAIN enhanced the FTS-5 score and 4m gait speed test (*p* < 0.05).

### 3.4. Effects of Frailty Status in Training and Detraining Effects on Functional Capacity and Frailty Level

**Figure 3:**
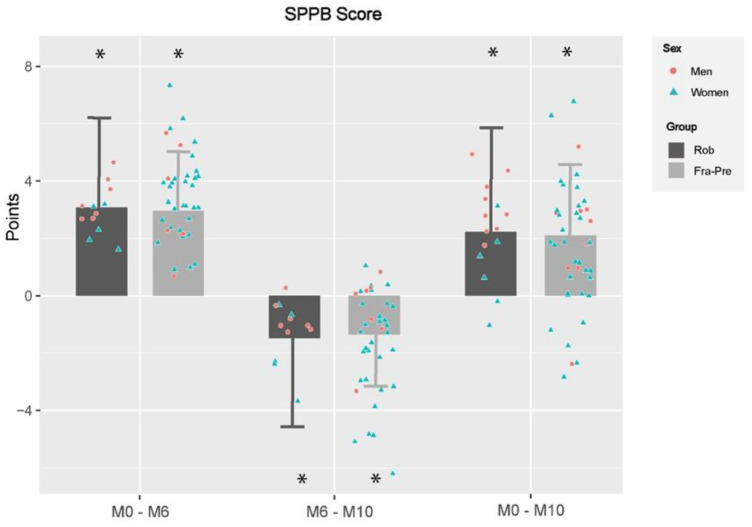
Changes in functional capacity between and within training subgroups in different evaluation periods. M0–M6: changes between baseline and 6th month; M6–M10: changes between 6th and 10th month; M0–M10: changes between baseline and 10th month; SPPB: Short Physical Performance Battery; PRE-FRA: Prefrails and frails participants of training group; ROB: Robust participants of training group; TRAIN: training group; *: significant differences within groups changes; Differences were obtained by linear mixed models adjusting by baseline values, gender and age; Statistical significance was set at *p* < 0.05.

**Figure 4:**
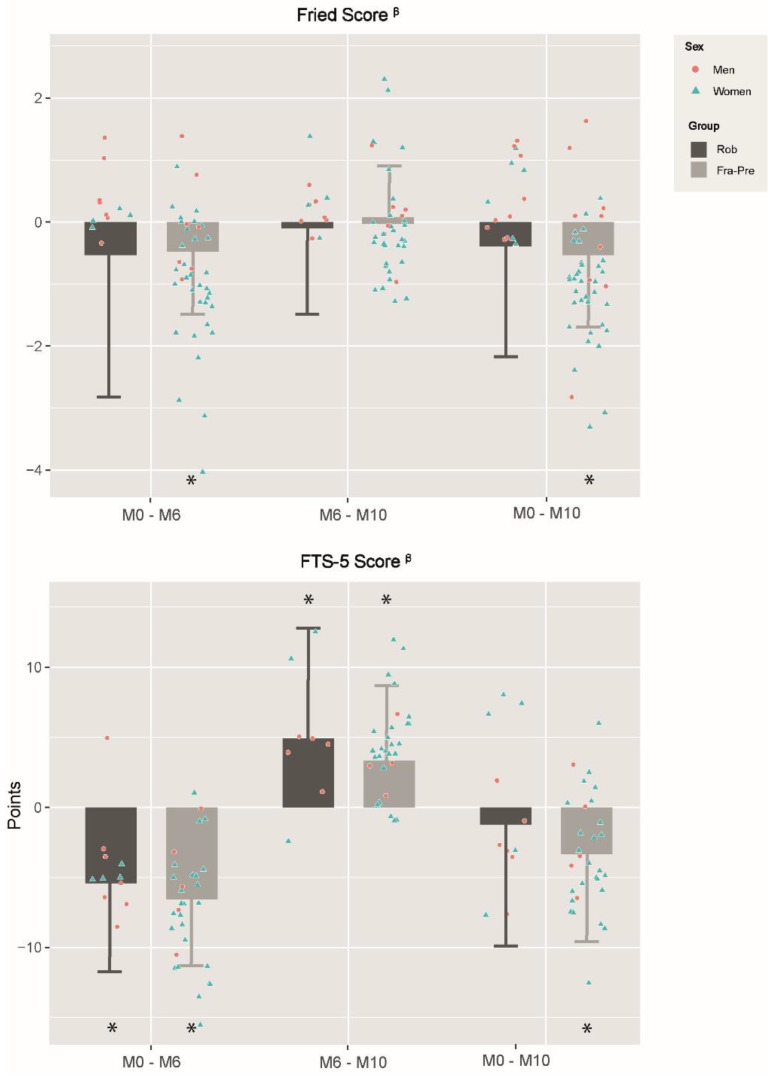
Changes in frailty levels between and within training subgroups in different evaluation periods. M0–M6: changes between baseline and 6th month; M6–M10: changes between 6th and 10th month; M0–M10: changes between baseline and 10th month; FTS-5: Frailty Trait Scale of 5 items; PRE-FRA: Prefrails and frails participants of training group; ROB: Robust participants of training group; TRAIN: training group; *: significant differences within groups changes; ^β^: negative changes represent frailty diminution; Differences were obtained by linear mixed models adjusting by baseline values, gender and age; Statistical significance was set at *p* < 0.05.

Changes obtained by different TRAIN subgroups according to their frailty status (FRA-PRE [mean age: 82.4 ± 5.6 y.] vs. ROB [77.1 ± 6.1 y.]; *p* < 0.05) are shown in Figure 3; Figure 4 and Table 6.

Regarding functional capacity (Figure 3), training effects were similar after 6-month MCT (M0–M6) since both subgroups improved in SPPB score and in every single test of the battery (all *p* < 0.001) (Table 6). With respect to detraining effects (M6–M10), there was an impairment in both subgroups in the SPPB score and on each test of the battery (all *p* < 0.05), except in the chair stand test for the ROB and in Romberg test for the FRA-PRE. Group effects were observed in Romberg test (all *p* < 0.05), unfavorable for the ROB. Nevertheless, despite the declines observed after detraining, both TRAIN subgroups improved with regard baseline in SPPB score and in every single test of the battery (all *p* < 0.05), except for the ROB in Romberg test. No group effect was observed.

Changes in frailty levels caused by training adaptations (M0–M6) are shown in Figure 4. While only FRA-PRE improved significantly in the Fried Phenotype (−0.5 ± 1.1), both subgroups enhanced in FTS-5 and its specific tests of Romberg test and 4m gait speed test (both *p* < 0.05) (Table 6). Moreover, FRA-PRE participants also improved in grip strength (*p* < 0.05). Any group effect was observed during this period. Regarding detraining adaptations (M6–M10), both subgroups worsened in the FTS-5. In addition, whereas both subgroups declined in the PASE, ROB also decreased in the Romberg test and FRA-PRE in the 4m gait speed test (*p* < 0.05). Nevertheless, no group effects were found. When the post-detraining evaluation was compared with baseline values (M0–M10), while FRA-PRE decreased the frailty score in the Fried and FTS-5 scales, ROB did not improve in any of them. Moreover, while FRA-PRE was also enhanced in the Romberg test, 4m gait speed and grip strength, ROB only improved in 4m gait speed test. Group effects favorable to FRA-PRE were found in the Romberg test.

## 4. Discussion

The main findings of the present study are: (1) Eelder-fit improves the functional capacity and frailty level of TRAIN participants, while CON suffered a decline in frailty assessed by FTS-5; (2) a 4-month detraining period leads to a drop in functional capacity and frailty evaluated through FTS-5 in TRAIN participants; (3) it seems that frailty status does not have a great influence in training and detraining adaptations on functional capacity and frailty level.

### 4.1. Training Effects on Functional Capacity

Eelder-fit has been shown to be effective in improving the functional capacity of older adults with or at risk of frailty. These results are in line with previous systematic reviews that have reported that MCT programs are, up to now, the best exercise strategy for improving functional outcomes in this population [35,37,38].

The improvements achieved in SPPB by our TRAIN (3.2 ± 2.4 pt) are in accordance with previous studies with frail individuals. Nevertheless, only Losa-Reyna et al. [39] reported similar enhancements (3.0 ± 1.5 pt.), being the rest inferior [18,40]. These results are highly relevant since it has been considered that a meaningful change in the SPPB ranges between 0.99 and 1.34 pt in this population [41]. On the other hand, and contrary to previous studies in which CON worsened [18,39,40], in our study they improved the score after the 6 months. This variation may be multifactorial but could partially be explained by the positive effect of the health-related talks performed during the study. Additionally, the increase in performance could also be produced by the cumulative repetition of the tests along evaluations, since the SPPB was also evaluated at the middle of the training program (3-month training). Despite the above, group effects were found in the SPPB and in every single test of the battery.

It seems that exercise programs lasting over 5-month may have better outcomes [42]. Accordingly, the better results obtained in our study with respect to the previous could be partially explained by the larger duration of Eelder-fit compared to most of them, since only the MCT program of Tarazona-Santabalina lasted 6 months [40]. Additionally, unlike other studies, the Eelder-fit protocol included functional training in the most advanced phases of training periodization. In these sessions, older adults performed exercises consisting of dynamic movements that simulated specific activities of daily living (ADL). Moreover, given the usual heterogeneity of physical function among older adults, previous studies have recommended focusing on personal skills to achieve optimal stimulus [18,43]. In this way, Eelder-fit was individualized and adapted depending on the functional capacity and individual toleration of the participants, ensuring a progressive and safe adaptation.

In conclusion, since gait speed, strength and dynamic balance can predict accelerated functional decline, ADL difficulty, falls, disability and mortality in older adults [44,45,46]; the improvements in functional parameters promoted by Eelder-fit are especially relevant, as they could prevent disability and adverse outcomes and consequently reduce health care-associated costs [16].

### 4.2. Training Effects on Frailty Level

In relation to frailty, our TRAIN led to a decrease in FP score, as previous studies with MCT interventions have shown [39,40]. However, the change obtained by our TRAIN (−0.7 ± 1.3) was inferior to that obtained by Losa-Reyna et al. [39] (−1.6 pt) and Tarazona-Santabalina et al. [40] (−2.0 pt). Given the ceiling effect of FP, these differences could be partially explained by the lower baseline punctuation obtained by our TRAIN in FP (1.5 ± 1.2) compared with those studies (3.1 ± 1.1 and 3.6 ± 0.8, respectively). In this regard, García-García et al. [10] concluded that FP shows some difficulties in assessing small changes in the elderly individual status, being this especially relevant in our study given the baseline FP score of the participants.

On the other hand, our TRAIN lowered the frailty score assessed by FTS-5. Given that it has emerged as a tool for the diagnostic of frailty in recent years, no comparable studies with exercise interventions have been found in the literature. Consequently, the results of the individual components evaluated within it will be analyzed separately. Our TRAIN improved in all single parameters of FTS-5, except in BMI, in which CON did not show relevant changes, as other studies reported [18,40]. The absence of changes in BMI of TRAIN may be due to the sample size, since the report of Moradell et al. [47] performed with the same cohort but with a bigger sample, showed a relevant decrease of BMI accompanied by a significant reduction of body fat percentage of TRAIN, whereas CON did not show changes. Turning to the present study, BMI was the only variable together with grip strength in which there was no significant group effect. Nevertheless, in the latter, TRAIN showed a significant improvement after training, in line with Losa-Reyna et al. [39] and contrary to Arrieta et al. [18], which did not obtain a relevant change. Furthermore, while TRAIN increased their PA registered throughout PASE, CON suffered a reduction, as the study by Losa-Reyna et al. [39] has also shown.

These findings are highly relevant given the relationship between frailty and numerous adverse events, including falls and fractures, cognitive decline, disability, hospitalization, nursing home placement, and death [48].

### 4.3. Detraining Effects on Functional Capacity

It is very common for older adults to have to stop exercise programs due to surgical operations, holiday periods, home-confinements due to COVID-19, pain or others. In this way, there are some studies have analyzed the impact of detraining on the physical fitness of this population [21,49,50]. Nevertheless, to the best of our knowledge, only a few reports have previously assessed the consequences of detraining on functional capacity [23,24].

After the 4-month detraining period, there was a worsening SPPB score (−1.2 ± 2.7 pt.) and every single test of the battery of the TRAIN, whereas the CON only declined in the chair stand test (all *p* < 0.05). Previous studies also found a decrease in SPPB scores after a 4- and 6-month detraining period following an MCT program of 8 and 6 months, respectively [23,24]. The negative changes in TRAIN could indicate that the functional gains achieved during the training period cannot be retained for a long time after activity cessation. It is worth noting that previous studies have concluded that a reduction of 1 pt in SPPB increases the risk of suffering adverse outcomes [51].

Hence, future studies should focus on exercise programs that reduce the negative effects of exercise interruptions. In this way, it could be beneficial to set shorter break periods or include an unsupervised training prescription during vacation periods [52]. Nonetheless, in this study, both groups presented higher SPPB scores when post-detraining values were compared with pre-training.

### 4.4. Detraining Effects on Frailty

While CON did not show any change, TRAIN held the performance in FP, although they did not maintain training gains in FTS-5 until the end of the detraining period, showing unfavorable group effects (*p* < 0.05). To date, the only study that has examined the effects of detraining on frailty has found a deterioration in TRAIN after the same period of detraining, although it used the Tilburg Frailty Indicator [53]. It is highly relevant, since increasing frailty scores over time is associated with an increased risk of adverse outcomes compared with maintaining or reducing them [54]. In our study, the different results obtained in TRAIN by both evaluation tools (FP vs. FTS-5) highlight the importance of conducting more studies comparing them. In this way, previous studies reported that FTS-5 presents a better capacity to monitor the evolution in elderly individuals, being of greater importance given that frailty is a continuous, unstable and revocable process [10]. As mentioned above, the performance maintenance of CON could be related to the positive effects of health-related talks.

Despite the previous declines in TRAIN, the values obtained at the end of detraining were better than at baseline in both scales, although no group effects were observed in any of the scales.

### 4.5. Effects of Frailty Status on Training and Detraining Effects on Functional Capacity and Frailty Level

Focusing on the effects of frailty status on exercise, non-group effects were found between TRAIN subgroups after 6-month training on functional capacity or frailty. In relation to this, previous studies have concluded that older adults with a lower degree of frailty may be able to train harder with respect to those at advanced stages of frailty [42]. Moreover, other research reports that those with the worst functional status at baseline have more possibilities to be non-responders to the exercise [55].

Regarding detraining, any differences between TRAIN subgroups were found in SPPB, FP and FTS-5. However, ROB obtained a greater performance drop in balance than FRA-PRE (*p* < 0.05).

Thus, based on this scenario, further research with greater samples is needed to identify and characterize those older adults with greater difficulties responding to the effects of training that could be more affected by detraining to provide them with the optimal exercise dose.

### 4.6. Strengths and Limitations

This study presents some limitations. First, even though the sample size was calculated a priori for the main comparison of the study, the secondary analysis of TRAIN subgroups presented a small and unbalanced sample (15 ROB vs. 45 FRA-PRE), avoiding the establishment of three subgroups (robust, prefrail and frail). This could have led to low statistical power in this comparison. Second, there was no randomization of the sample because of pragmatic (to maximize training attendance) and ethical reasons, since not prescribing exercise to older adults may be considered unethical [56]. In addition, this condition simulates real-life conditions, where motivated people do exercise and unmotivated people do not. Despite this, heigh was the only variable that presented differences between CON and TRAIN at baseline.

On the contrary, the present study has several strengths. This is one of the first studies to evaluate detraining adaptations to functional capacity and frailty in older adults with or at risk of frailty. Furthermore, no research has previously focused on analyzing the effects of frailty status on training and detraining adaptations above the same variables. Moreover, the exercise program was individualized according to the functional capacity and individual abilities of the participants, which could help to develop tailored and individualized protocols for this population. Finally, the training protocol and methodology have been described with accuracy so that it can be easily replicated.

## 5. Conclusions

In conclusion, Eelder-fit has proved to be feasible and beneficial in older adults with or at risk of frailty, showing positive effects on the functional capacity and frailty levels of this population. Furthermore, 4-months of detraining caused a drop of these variables, except for Fried Phenotype. In order to avoid reversibility of the benefits gained with exercise programs, it could be beneficial to promote ongoing physical programs, encouraging smaller break periods or implementing them with an unsupervised exercise prescription. Moreover, it seems that frailty status does not have a great influence on training and detraining adaptations above functional capacity and frailty levels.

## Figures and Tables

**Table 1:** Eelder-fit training periodization.

**Strength & Power Session Periodization**	**Phase**	**PHASE 1** **Familiarization (Weeks 1–4)**				**PHASE 2** **Strength (Weeks 5–14)**										**PHASE 3** **Coordination and Power (Weeks 15–21)**							**PHASE 4** **Functional and Power** **(Weeks 22–24)**		
	**Goals**	Cause training adaptations				Increase strength levels										Enhance intermuscular coordination				Increase power			Improve performance DLA		
		Learn technical executions				Increase muscle endurance										Increase muscle endurance and strength level				Increase strength levels			Increase power and coordination		
	**Weeks**	1	2	3	4	5	6	7	8	9	10	11	12	13	14	15	16	17	18	19	20	21	22	23	24
	Type of session	ST	ST	ST	ST	ST	ST	ST	ST	ST	ST	ST	ST	ST	ST	ST	ST	ST	PW	PW	PW	PW	PW	PW	PW
	Sessions/week	2	2	2	2	2	2	2	2	2	2	2	2	2	2	2	2	2	2	2	2	2	1	1	1
	Nº Ex *	6(2)	6(2)	7(2)	7(2)	7 ●(2)	7(2)	7(2)	8 ‡(2)	8 ●(2)	8(2)	8 ‡(2)	8 ●(2)	8(2)	8 ‡(2)	7 ●(2)	7	7 ●	7	6 ●(6)	6 ‡(6)	7(7)	6 ●(6)	6 ‡(6)	6(6)
	Sets	1	2	2	2	2	2	2	2	2	2	2	2	2	2	2	2	2	2	2	2	2	2	3	3
	Rep & Speed	8↓	8↓	10↓	10↓	10→	12→	15→	12→	12→	15→	12→	12→	15→	12→	12→	15→	12→	15→	12↑	12↑	15↑	12↑	12↑	15↑
	Balance ex (s)	15	15	20	20	30	30	30	30	30	30	30	30	30	30	30	-	-	-	20	20	20	30	30	30
	Set Rest time (s)	90	90	90	90	60	60	60	60	60	60	60	60	60	60	75	75	75	75	90(20*_a_*)	90(20*_a_*)	90(20*_a_*)	90(30*_a_*)	90(30*_a_*)	90(30*_a_*)
**Aerobic Endurance & Functional Session Periodization**	**Phase**	**PHASE 1** **Familiarization (Weeks 1–4)**				**PHASE 2** **Development (Weeks 5–14)**										**PHASE 3** **Maintenance (Weeks 15–21)**							**PHASE 4#** **Functional and Power** **(Weeks 22–24)**		
	**Goals**	Increase aerobic capacity (VO_2_ max)				Increase aerobic capacity (VO_2_ max)										Increase aerobic capacity (VO_2_ max)							Improve performance DLA		
		Improve coordination and functional performance				Improve coordination and functional performance										Improve coordination and functional performance							Increase power and coordination		
		Enhance motor skills and dynamic balance				Enhance motor skills and dynamic balance										Enhance motor skills and dynamic balance									
	**Weeks**	1	2	3	4	5	6	7	8	9	10	11	12	13	14	15	16	17	18	19	20	21	22	23	24
	Type of session	AE	AE	AE	AE	AE	AE	AE	AE	AE	AE	AE	AE	AE	AE	AE	AE	AE	AE	AE	AE	AE	FUN	FUN	FUN
	Sessions/week	1	1	1	1	1	1	1	1	1	1	1	1	1	1	1	1	1	1	1	1	1	2	2	2
	Nº Ex	7	7	7	7	7 ●	7	7	7‡	7 ●	7	7	7 ‡	7 ●	7	7	7 ‡	7 ●	7	7	7 ‡	7	6	6	6
	Sets	2	2	2	2	2	2	2	2	2	2	2	2	2	2	2	2	2	2	2	2	2	2	2	2
	Set time (s)	30	30	45	45	60	60	60	60	75	75	75	75	90	90	90	90	90	90	90	90	90	60	75	90
	Set Rest time (s)	60	60	90	90	90	90	75	75	75	75	60	60	60	60	90(30*_b_*)	90(30*_b_*)	90(45*_b_*)	90(45*_b_*)	90(60*_b_*)	90(60*_b_*)	90(60*_b_*)	60(30_a_)	75(45_a_)	90(60_a_)
	Total WTs	7	7	10.5	10.5	14	14	14	14	17.5	17.5	17.5	17.5	21	21	28	28	31.5	31.5	35	35	35	18	24	30
	Ratio (WT:RT) (s)	1:2	1:2	1:2	1:2	1:1.5	1:1.5	1:1.25	1:1.25	1:1	1:1	1.25:1	1.25:1	1.5:1	1.5:1	2:1	2:1	2.25:1	2.25:1	2.5:1	2.5:1	2.5:1	1.5:1	2:1	2.5:1

Note: ↓: low speed execution (concentric and eccentric phase in approximately 4s); →: moderate speed execution (concentric and eccentric phase in approximately 2s); ↑: high speed execution (executed as fast as possible during the concentric phase, followed by a controlled eccentric phase of approximately 2 s); ●: exercises change; ‡: overload. *: number of balance exercises are between brackets; *_a_:* balance exercises performed during the active rest of power and functional session; *_b_*_:_ static and dynamic balance exercises and coordination tasks using ball and balloon handling performed during the active rest of aerobic endurance sessions; #: this phase correspond to the 4th phase of strength periodization; AE: Aerobic endurance sessions; ADL: activities of daily living; Ex: exercises; FUN: functional sessions; Rep & Speed: repetitions and speed execution; PW: power sessions; ST: strength sessions; WTs: Total Work time session excluding 10–15 min warm up (joint mobility, balance and cardiorespiratory exercises were performed), and a 10–15 min cool down (flexibility exercises and cognitive tasks).

**Table 2:** Eelder-fit methodology–protocol.

	**Weeks**	1	2	3	4	5	6	7	8	9	10	11	12	13	14	15	16	17	18	19	20	21	22	23	24
**STRENGTH, POWER & FUNCTIONAL** **TRAINING** **METHODOLOGY**	**Phase**	**PHASE 1** **Familiarization (Weeks 1–4)**				**PHASE 2** **Strength (Weeks 5–14)**										**PHASE 3** **Coordination and Power (Weeks 15–21)**							**PHASE 4** **Functional and Power** **(Weeks 22–24)**		
	**Goals**	Cause training adaptations				Increase strength levels										Enhance intermuscular coordination				Increase power			Improve performance DLA		
		Learn technical executions				Increase muscle endurance										Increase muscle endurance and strength level				Increase strength levels			Increase power and coordination		
	**Equipment**	Elastic resistance bands, free weights (dumbbells, weighted anklets and medicine balls) and fitballs																							
	**Strength and Power Exercises**	Exercises involving large muscle groups through single movements of lower or upper limbs				Exercises involving large muscle groups through single movements of lower or upper limbs										Exercises involving large muscle groups combined multi-joint movements of lower and upper limbs				Exercises involving large muscle groups through single movements of lower or upper limbs			Exercises involving large muscle groups combined multi-joint movements of lower and upper limbs		
		Light weights lifted at low speed				Medium and heavier weights lifted at moderate speed														Medium weights lifted at fast as possible in the concentric phase					
		Performed exercises: Chest press and fly, shoulder press, flexion and abduction, triceps pushdown, kickbacks and overhead extensions, biceps curl, pull-down, high and low back row, pull apart, lower-back extension, trunk rotation, abdominal crunch through sit position, different types of squats, quadriceps extension, leg curl, hip abduction, adduction, flexion and extension and calf raise																							
	**Balance Exercises**	Static balance exercises				Static balance exercises decreasing limb involvement, base support and input of information from the senses in addition to induce variations in the center of gravity										Balance training included in the strength exercise executions				Static balance exercises decreasing limb involvement, base support and input of information from the senses in addition to induce variations in the center of gravity					
		Double leg stance with feet together, single leg stances, semi-tandem and tandem stance				Double leg stance with feet together, single leg stance, semi-tandem and tandem stance with or without the movement of some objects or parts of the body														Double leg stance with feet together, single leg stance, semi-tandem and tandem stance with or without the movement of some objects or parts of the body					
	**Functional Exercises**																						Exercises consisting of dynamic movements that simulated ADL		
																							Shopping, walking avoiding obstacles, bringing and serving food and drink, climbing up and down stairs, walking fast to “take the public transport” and getting up from the floor		
	**Weeks**	1	2	3	4	5	6	7	8	9	10	11	12	13	14	15	16	17	18	19	20	21	22	23	24
**AEROBIC ENDURANCE** **TRAINING** **METHODOLOGY**	**Phase**	**PHASE 1** **Familiarization (weeks 1–4)**				**PHASE 2** **Development (weeks 5–14)**										**PHASE 3** **Maintenance (weeks 15–21)**									
	**Goals**	Increase aerobic capacity				Increase aerobic capacity										Increase aerobic capacity									
		Improve coordination and functional performance				Improve coordination and functional performance										Improve coordination and functional performance									
		Enhance motor skills and dynamic balance				Enhance motor skills and dynamic balance										Enhance motor skills and dynamic balance									
	**Equipment**	Psychomotricity material, agility ladders, static cycles, steps, dumbbells, weighted anklets, balls and balloons.																							
	**Aerobic Exercises**	Basic exercises with an increase in speed or frequency. The load was also increased by hardening the resistance level in cycling or including slight free weights while performing exercises																							
		Walking, step exercises and stationary cycle for legs, arms or both.																							
	**Dynamic Balance-Agility Exercises**	Difficulty progressively increased involving both motor (perturbing the center of gravity throughout different types of displacement, changes of direction and/or velocity), load (including slight free weights while performing exercises) and cognitive tasks (dual- and multi-task activities)																							
		Walking with change of direction, toe and heel walking, tandem gait, and																							
	**Motor and Coordination Skills**	Eye-Hand, Eye-Leg or Eye-Hand-Leg coordination. Difficulty should progressively increase involving both motor and cognitive tasks (dual- and multi-task activities)																							
		Static or dynamic skills-handling with ball (bounce, passes and receptions, throws, turns, changes of direction) and balloon (keep control of the balloon with hands and/or legs)																							

Note: ADL: activities of daily living.

**Table 3:** Characteristics of CON and TRAIN at baseline.

Characteristics	Whole Sample (*n* = 106)	Control (*n* = 46)	Training (*n* = 60)	*p* Value CON vs. TRAIN
**Age (years)**	80.5 ± 6.0	79.7 ± 5.8	81.1 ± 6.2	0.216
**Sex**				
Males	25 (29.1)	9 (19.6)	19 (31.7)	0.161
Females	63 (70.9)	37 (80.4)	41 (68.3)	
**Functional capacity & ADL performance**				
SPPB (p)	7.7 ± 1.7	7.8 ± 1.7	7.5 ± 1.8	0.389
IADL score	10.2 ± 4.1	10.1 ± 3.8	10.3 ± 4.4	0.858
Barthel Index score	95.5 ± 7.3	95.0 ± 8.4	96.0 ± 6.4	0.515
**Frailty level**				
Fried (p)	1.6 ± 1.3	1.6 ± 1.2	1.5 ± 1.3	0.828
n robusts (fried criteria)	23 (21.7)	8 (17.4)	15 (25.0)	
n pre-frails (fried criteria)	73 (68.9)	33 (71.7)	40 (66.7)	
n frails (fried criteria)	10 (9.4)	5 (10.9)	5 (8.3)	
FTS-5 (p)	18.8 ± 6.9	18.3 ± 7.3	19.1 ± 6.7	0.612
Physical Activity and Sedentary Behaviour *				
ST & SB (min/day)	1333.5 ± 66.1	1334.1 ± 64.0	1331.5 ± 63.1	0.915
LPA (min/day)	89.4 ± 51.8	89.7 ± 52.0	93.6 ± 52.4	0.954
MVPA (min/day)	17.1 ± 20.7	16.2 ± 19.8	14.9 ± 15.4	0.625
**Body composition measurement**				
BMI	29.6 ± 5.3	29.5 ± 5.5	29.8 ± 5.1	0.821
Weight (kg)	72.4 ± 13.6	69.5 ± 13.0	74.4 ± 13.8	0.077
Height	155.6 ± 10.4	152.8 ± 11.8	157.7 ± 8.7	0.017
% BF	37.7 ± 6.5	38.3 ± 6.6	37.3 ± 6.5	0.478
**Cognitive impairment**				
Minimental score	25.8 ± 4.2	25.8 ± 4.5	25.63 ± 4.7	0.957
**Malnutrition**				
MNA	24.4 ± 3.4	24.7 ± 3.1	24.0 ± 4.7	0.277

Number of participants of the sample n and (%) per group for categorical variables; mean and standard deviation (S.D.) for continuous variables. SPPB: Short Physical Performance Battery; FTS-5: Frailty Trait Scale of 5 items; ST & SB: sedentary time and sedentary behavior; LPA: light intensity physical activity; MVPA: moderate to vigorous physical activity; p: points; BMI: Body Mass Index; % BF: body fat percentage; IADL instrumental activities of daily living; MNA: Mini Nutritional Assessment *: adjusted by 24 valid hours; **Boldface** indicates significant results, which were obtained using Student’s *t*-test and Chi-square test for continuous and categorical data, respectively. Statistical significance was set at *p* < 0.05.

**Table 4:** Changes in functional capacity between and within groups in different evaluation periods.

	Post-Training vs. Pre-Training M0–M6					Post-Detraining vs. Post-Training M6–M10					Post-Detraining vs. Pre-Training M0–M10				
	CON (*n* = 35)		TRAIN (*n* = 51)		Group Effects	CON (*n* = 27)		TRAIN (*n* = 49)		Group Effects	CON (*n* = 31)		TRAIN (*n* = 56)		Group Effects
	Change	*p* Value	Change	*p* Value	*p* Value	Change	*p* Value	Change	*p* Value	*p* Value	Change	*p* Value	Change	*p* Value	*p* Value
Romberg test (pt)	−0.3 ± 1.28	**0.021**	0.7 ± 1.1	**<0.001**	**<0.001**	−0.4 ± 2.08	0.084	−0.4 ± 1.4	**0.013**	0.970	−0.3 ± 1.9	0.164	0.1 ± 1.4	0.721	0.160
4-m Gait speed test (s) ^β^	−0.4 ± 1.7	**0.036**	−1.5 ± 1.4	**<0.001**	**<0.001**	0.2 ± 2.11	0.532	0.6 ± 1.5	**<0.001**	0.125	−0.3 ± 2.2	0.248	−1.0 ± 1.6	**<0.001**	**0.009**
Chair stand test (s) ^β^	−4.0 ± 5.5	**<0.001**	−6.6 ± 4.3	**<0.001**	**<0.001**	1.1 ± 4.5	0.033	1.5 ± 3.2	**<0.001**	0.129	−3.4 ± 5.4	**<0.001**	−4.8 ± 3.8	**<0.001**	**0.046**

Note: Change: Mean ± standar deviation. M0–M6: changes between baseline and 6th month; M6–M10: changes between 6th and 10th month; M0–M10: changes between baseline and 10th month; CON: Control Group; TRAIN: Training group; SPPB: Short Physical Performance Battery; ^β^: negative changes represent performance improvement; pt: points; **Boldface** indicates significant results, which were obtained by linear mixed models adjusting by baseline values, gender and age. Statistical significance was set at *p* < 0.05. When post-training evaluation is compared with post-detraining (M6–M10), TRAIN suffered a significant decline in SPPB score (−1.2 ± 2.7; *p* < 0.001) and in each test of the battery, while CON worsened in the chair stand test (all *p* < 0.05). Even though the previous declines, both groups improved their SPPB scores and chair stand tests when pre-training values were compared with post-detraining (M0–M10). In addition, TRAIN participants also improved in 4m gait speed (all *p* < 0.05). Group effects were found in the last and in the chair stand test, being favorable to TRAIN.

**Table 5:** Changes in FTS-5 tests between and within groups in different evaluation periods.

	Post-Training vs. Pre-Training M0–M6					Post-Detraining vs. Post-Training M6–M10					Post-Detraining vs. Pre-Training M0–M10				
	CON (*n* = 26)		TRAIN (*n* = 41)		Group Effects	CON (*n* = 17)		TRAIN (*n* = 38)		Group Effects	CON (*n* = 17)		TRAIN (*n* = 41)		Group Effects
	Change	*p* Value	Change	*p* Value	*p* Value	Change	*p* Value	Change	*p* Value	*p* Value	Change	*p* Value	Change	*p* Value	*p* Value
Romberg test (FTS-5 score) ^β^	1.7 ± 4.8	**0.005**	−2.2 ± 3.7	**<0.001**	**<0.001**	0.7 ± 8.2	0.518	1.6 ± 4.8	**0.014**	0.457	−0.1 ± 7.3	0.940	−0.5 ± 4.6	0.425	0.693
BMI (kg/m^2^)	−0.3 ± 1.8	0.200	−0.1 ± 1.4	0.628	0.433	0.3 ± 1.1	0.100	0.1 ± 0.7	0.415	0.292	−0.1 ± 2.3	0.756	−0.3 ± 1.4	0.183	0.637
PASE (pt)	−22.7 ± 65.6	**0.006**	16.1 ± 50.2	**0.010**	**<0.001**	−14.2 ± 69.42	0.132	−25.2 ± 41.8	**<0.001**	0.295	−16.4 ± 67.7	0.067	−9.6 ± 41.8	0.082	0.481
4-m Gait speed test (s) ^β^	−0.3 ± 1.8	0.139	−1.6 ± 1.4	**<0.001**	**<0.001**	−0.1 ± 2.0	0.786	0.7 ± 1.3	**<0.001**	**0.009**	−0.3 ± 2.7	0.410	−1.1 ± 1.6	**<0.001**	0.051
Grip strength (kg)	1.3 ± 6.2	0.085	2.7 ± 5.4	**<0.001**	0.102	0.5 ± 4.5	0.413	0.9 ± 3.4	0.060	0.570	2.0 ± 7.3	**0.037**	3.1 ± 5.1	**<0.001**	0.289

Note: Change: Mean ± standard deviation. M0–M6: changes between baseline and 6th month; M6–M10: changes between 6th and 10th month; M0–M10: changes between baseline and 10th month; FTS-5: Frailty Trait Scale of 5 items; BMI: body mass index; pt: points; ^β^: negative changes represent performance improvement; CON: Control Group; TRAIN: Training group; **Boldface** indicates significant results, which were obtained by linear mixed models adjusting by baseline values, gender, and age. Statistical significance was set at *p* < 0.05.

**Table 6:** Changes in specific tests of SPPB and FTS-5 at different time points in the training subgroups.

**SPPB**		**Post-Training vs. Pre-Training** **M0–M6**					**Post-Detraining vs. Post-Training** **M6–M10**					**Post-Detraining vs. Pre-Training** **M0–M10**				
		**ROB (*n* = 12)**		**FRA-PRE (*n* = 39)**		**Group Effects**	**ROB (*n* = 12)**		**FRA-PRE (*n* = 37)**		**Group Effects**	**ROB (*n* = 15)**		**FRA-PRE (*n* = 41)**		**Group Effects**
		**Change**	***p* Value**	**Change**	***p* Value**	***p* Value**	**Change**	***p* Value**	**Change**	***p* Value**	***p* Value**	**Change**	***p* Value**	**Change**	***p* Value**	***p* Value**
	Romberg test (pt)	0.8 ± 1.1	**<0.001**	0.8 ± 0.7	**<0.001**	0.943	−1.1 ± 2.0	**<0.001**	−0.3 ± 1.3	0.106	**0.034**	−0.1 ± 1.9	0.591	0.4 ± 1.3	**0.016**	0.089
	4-m Gait speed test (s) ^β^	−1.8 ± 2.1	**<0.001**	−1.6 ± 1.3	**<0.001**	0.625	0.7 ± 2.2	**0.034**	0.6 ± 1.4	**0.007**	0.767	−1.1 ± 2.1	**<0.001**	−1.1 ± 1.4	**<0.001**	0.954
	Chair stand test (s) ^β^	−6.7 ± 7.3	**<0.001**	−6.7 ± 4.6	**<0.001**	0.949	0.4 ± 4.7	0.539	2.0 ± 3.1	**<0.001**	0.077	−5.9 ± 6.0	**<0.001**	−4.6 ± 4.0	**<0.001**	0.230
F**TS-5**		**ROB (*n* = 11)**		**FRA-PRE (*n* = 30)**		**Group Effects**	**ROB (*n* = 8)**		**FRA-PRE (*n* = 30)**		**Group Effects**	**ROB (*n* = 11)**		**FRA-PRE (*n* = 30)**		**Group Effects**
		**Change**	***p* Value**	**Change**	***p* Value**	***p* Value**	**Change**	***p* Value**	**Change**	***p* Value**	***p* Value**	**Change**	***p* Value**	**Change**	***p* Value**	***p* Value**
	Romberg test (SPPB score) ^β^	−2.4 ± 3.5	**<0.001**	−2.9 ± 2.5	**<0.001**	0.463	3.5 ± 6.8	**0.003**	1.1 ± 4.5	0.155	0.085	0.5 ± 6.3	0.619	−2.1 ± 4.5	**0.004**	**0.041**
	BMI (kg/m^2^)	0.1 ± 1.9	0.750	−0.2 ± 1.3	0.461	0.530	0.1 ± 1.1	0.703	0.1 ± 0.7	0.339	0.846	−0.4 ± 2.3	0.226	−0.1 ± 1.6	0.632	0.489
	PASE (pt)	20.5 ± 87.8	0.138	11.9 ± 60.3	0.209	0.623	−23.1 ± 69.9	**0.046**	−36.3 ± 46.2	**<0.001**	0.353	−6.6 ± 67.0	0.529	−12.2 ± 50.1	0.124	0.672
	4-m Gait speed test (s) ^β^	−1.8 ± 1.9	**<0.001**	−1.9 ± 1.3	**<0.001**	0.859	0.7 ± 2.2	0.059	0.7 ± 1.5	**0.004**	0.952	−1.2 ± 2.3	**0.002**	−1.2 ± 1.6	**<0.001**	0.951
	Grip strength (kg)	1.5 ± 7.4	0.207	2.8 ± 4.6	**<0.001**	0.303	1.7 ± 4.8	**0.037**	0.4 ± 3.4	0.431	0.169	2.0 ± 7.5	0.083	3.1 ± 4.9	**<0.001**	0.394

Note: Change: Mean ± standard deviation. M0–M6: changes between baseline and 6th month; M6–M10: changes between 6th and 10th month; M0–M10: changes between baseline and 10th month; ROB: Robust participants of training group; PRE-FRA: Prefrails and frails participants of training group; FTS-5: Frailty Trait Scale of 5 items; SPPB: Short Physical Performance Battery; pt: points; ^β^: negative changes represent performance improvement; **Boldface** indicates significant results, which were obtained by linear mixed models adjusting by baseline values, gender, and age. Statistical significance was set at <0.05.

## Data Availability

The data used in this study can be obtained upon request from the corresponding author.
